# RETRACTION: The Effects of Enhanced Recovery After Surgery on Wound Infection, Complications, and Postoperative Hospital Stay in Patients Undergoing Colorectal Surgery: A Systematic Review and Meta‐Analysis

**DOI:** 10.1111/iwj.70307

**Published:** 2025-03-06

## Abstract

**RETRACTION:** LiN. , WeiS. , QiY. , and WeiW. , “The Effects of Enhanced Recovery After Surgery on Wound Infection, Complications, and Postoperative Hospital Stay in Patients Undergoing Colorectal Surgery: A Systematic Review and Meta‐Analysis,” International Wound Journal 20, no. 10 (2023): 3990–3998, 10.1111/iwj.14287.37650448 PMC10681523

The above article, published online on 31 August 2023, in Wiley Online Library (http://onlinelibrary.wiley.com/), has been retracted by agreement between the journal Editor in Chief, Professor Keith Harding; and John Wiley & Sons Ltd. Following an investigation by the publisher, all parties have concluded that this article was accepted solely on the basis of a compromised peer review process. The editors have therefore decided to retract the article. The authors did not respond to our notice regarding the retraction.

**RETRACTION:** N. Li , S. Wei , Y. Qi , and W. Wei , “The Effects of Enhanced Recovery After Surgery on Wound Infection, Complications, and Postoperative Hospital Stay in Patients Undergoing Colorectal Surgery: A Systematic Review and Meta‐Analysis,” International Wound Journal 20, no. 10 (2023): 3990–3998, 10.1111/iwj.14287.37650448 PMC10681523

The above article, published online on 31 August 2023, in Wiley Online Library (http://onlinelibrary.wiley.com/), has been retracted by agreement between the journal Editor in Chief, Professor Keith Harding; and John Wiley & Sons Ltd. Following an investigation by the publisher, all parties have concluded that this article was accepted solely on the basis of a compromised peer review process. The editors have therefore decided to retract the article. The authors did not respond to our notice regarding the retraction.

